# 

**Published:** 1891-11

**Authors:** 


### Society Meetings.

THE SOUTHERN SURGICAL AND GYNECOLOGICAL ASSOCIATION will hold its fourth annual meeting in Richmond, Va., November 10, 11, and 12, 1891, under the presidency of Dr. Lewis S. McMurtry, of Louisville, Ky. This association is doing a most useful work for the profession in the South, in whose interest it was mainly organized. There is no grander medical society anywhere than this, and we bespeak for it a continued and lasting existence. The titles of the papers to be read in the forthcoming meeting indicate that it will be one of especial excellence. Members of the profession everywhere are cordially invited to attend. Dr. W. E. B. Davis, of Birmingham, Ala., is the secretary, and Dr. Hunter McGuire, of Richmond, is chairman of the committee of arrangements. These, together with the president, constitute a triad of scientific workers of which any society may well be proud.

### BOOKS AND PAMPHLETS RECEIVED.

A Text-Book of Physiology. By M. Foster, M. A., M. D., LL. D., F. R. S., Professor of Physiology in the University of Cambridge, and Fellow of Trinity College, Cambridge. Fourth American, from the Fifth English edition, thoroughly revised, with notes, additions, and two hundred and eighty-two illustrations. Octavo, pp. xvi.—1072. Philadelphia: Lea Brothers & Co. 1891.

Essentials of Bacteriology; being a Concise and Systematic Introduction to the Study of Microörganisms for the use of Students and Practitioners. By M. V. Ball, M. D., late Resident Physician German Hospital, Philadelphia; Assistant in Microscopy, Niagara University, Buffalo, N. Y., etc. With seventy-seven illustrations, some in colors. Saunders' Question Compends, No. 20. Philadelphia: W. B. Saunders, 913 Walnut street. 1891.

Artificial Anesthesia and Anesthetics. By DeForest Willard, R. M., M. D., Ph. D., Clinical Professor of Orthopedic Surgery in the University of Philadelphia; Surgeon to the Presbyterian Hospital, etc., and Lewis H. Adler, Jr., M. D., Instructor in Rectal Diseases, Philadelphia Polyclinic and College for Graduates in Medicine. The Physicians' Leisure Library. Single copies, cloth, 50 cents; paper, 25 cents. Detroit, Mich.: Geo. S. Davis. 1891.

Buffalo Society of Natural Sciences, Thirtieth Annual Meeting, June 12, 1891. Containing Addresses of the President, with reports of officers, committees, etc. Buffalo: Baker, Jones & Co., Printers and Binders. 1891.

The Snook-Herr Poisoning. The Official Investigation. Preliminary Examination. By H. M. Goodman, M. D., Demonstrator of Physiology, Bacteriology and Pathology, University of Louisville. Reprint: *American Practitioner and News*, April 25, 1891.

Resection as a Substitute for Primary Amputation. By Thomas H. Manley, M. D., New York City, Visiting Surgeon to Harlem Hospital, New York. Read before the Danbury (Ct.) Medical Society, January 4, 1891. Reprint: *New England Medical Monthly*.

Fractures at the Elbow Joint. By Thomas H. Manley, M. D. Reprint: *The New York Medical Journal*, June 27, 1891.

Tumors of the Naso-Pharynx, Pharynx, Larynx and Œsophagus. By W. Cheatham, M. D., Louisville, Clinical Lecturer on the Eye, Ear, Throat, and Nose, University of Louisville. Reprint: *The New York Medical Journal*, August 15, 1891.

The Administration of Guaiacol Iodide by the Intestines in the Treatment of Tuberculosis of the Lungs. By Wm. H. Gregg, M. D., New York. Reprint: *The New York Medical Journal*, September, 26, 1891.

Remarks on a Series of One Hundred Laparatomies. By Joseph Taber Johnson, A. M., M. D., Ph. D., Washington, D. C., Professor of Gynecology in the Medical Department, Georgetown University; Gynecologist to Providence Hospital; Vice-President of the American Gynecological Society; Fellow of the British Gynecological Society; President of the Medical Society of the District of Columbia in 1887; President Washington Obstetrical and Gynecological Society, 1888-1889; Fellow Southern Surgical and Gynecological Society; Gynecological Surgeon to Columbia Hospital. Reprint: *Virginia Medical Monthly*, October, 1891.

Trachoma and its Treatment. Powdered Jequirity. Written for the *Ophthalmic Record* by W. Cheatham, M. D., Clinical Lecturer on Diseases of the Eye, Ear, Nose, and Throat, University of Louisville.

The Study of Ophthalmic Therapeutics. By F. Park Lewis, M. D., Buffalo, N. Y. Reprint: *The Hahnemannian Monthly*, September, 1891.

My Personal Experience with Vaginal Hysterectomy. By Florian Krug, M. D., New York. Reprint: *The American Journal of Obstetrics and Diseases of Women and Children*. Vol. XXIV., No. 7, 1891.

Four Cases of Removal of Myomata by Abdominal Section. By James F. W. Ross, M. D., Lecturer on Abdominal Surgery, Toronto University; on Diseases of Women, Woman's Medical College; Gynecologists Toronto General Hospital, Toronto Dispensary, and St. John's Hospital for Women. Reprint: *American Journal of Obstetrics and Diseases of Women and Children*. Vol. XLV., No. 9, 1891.

### HEALTH BULLETINS.

Michigan Board of Health. September, 1891.

Newport, R. I., Reports of Deaths and Contagious Diseases. September, 1891.

Abstracts of Sanitary Reports, U. S. Marine Hospital Service. Vol. VI., Nos. 35 to 42.

WANTED—The Berlin Royal Library wishes to complete its file of the Buffalo Medical and Surgical Journal and advertises for Vols. I. to XXVIII. inclusive, of the new series, and the fifteen volumes of the old series. All inquiries on the subject of back numbers and prices should be addressed to the Managing Editor, 284 Franklin street, Buffalo, N. Y.

FOR SALE—Valuable Medical Library and Surgical Instruments for sale cheap. Address, Mrs. B. A. Fuller, LeRoy, N.Y.

---

Notice to Contributors. — We are glad to receive contributions from every one who knows anything of interest to the profession. Articles designed for publication in the Journal should be handed in before the first day of the month. The Editors are not responsible for the views or opinions of contributors. All communications should be addressed to the Managing Editor, 284 Franklin Street, Buffalo, N. Y.

